# Effect of Yijinjing exercise on cervical spondylosis: A protocol for systematic review

**DOI:** 10.1097/MD.0000000000020764

**Published:** 2020-07-02

**Authors:** Qian Zhuang, Huichao Feng, Fushi Jing, Jiao Rong, Yueming Lv, Fujie Jing, Jing Zhang

**Affiliations:** a School of Acupuncture-Tuina, Shandong University of Traditional Chinese Medicine, Jinan; b Department of Rehabilitation, The People's Hospital of Juxian, Juxian; c Laboratory management office, Shandong University of Traditional Chinese Medicine, Jinan, Shandong, China.

**Keywords:** cervical spondylosis, protocol, systematic, Yijinjing

## Abstract

**Background:**

Cervical spondylosis (CS) is a common disease due to the modern lifestyle. Yijinjin, a kind of traditional Chinese exercise, is frequently used for the prevention of CS by Traditional Chinese Medicine doctors. However, there is no relevant systematic review show its effectiveness and safety. The study aims to evaluate the effectiveness and safety of Yijinjing for patients with CS.

**Methods:**

The following electronic databases will be searched from the respective dates of database inception to June 1st, 2020: The Cochrane Library, Web of Science, Springer, EMBASE, MEDLINE, the World Health Organization International Clinical Trials Registry Platform, the Chinese Biomedical Literature Database, China National Knowledge Infrastructure, Wanfang database, the Chinese Scientific Journal Database, and other sources. All published randomized controlled trials (RCTs) and blinded researches that are relevant to the subject of interest only will be contained. Two independent researchers will operate article retrieval, duplication removing, screening, quality evaluation, and data analyses by Review Manager (V.5.3.5). Meta-analyses, subgroup analysis and/or descriptive analysis will be performed based on the included data conditions.

**Results:**

High-quality synthesis and/or descriptive analysis of current evidence will be provided from the neck disability index, neck pain questionnaire questionnaires, patient satisfaction scale and adverse reactions.

**Conclusion:**

This study will provide the evidence of whether Yijinjing is an effective and safe intervention for people with CS.

**PROSPERO registration number:**

CRD42020164706.

## 1 Introduction

### 1.1 Description of the condition

Cervical spondylosis (CS) is cervical intervertebral disc degenerative change and its adjacent structure of secondary pathological changes involved around organizational structure. CS seriously affects the quality of life for its heavy disease burden. Epidemiological investigation shows that the incidence of CS is increasing and getting younger year by year.^[1]^ The etiology and pathogenesis of CS is not entirely clear, generally considered to be the result of joint action of many factors. The following factors is related to CS: age, sex, occupation, education levels, area, income, BMI, life style (e.g., smoking, drinking, exercise frequency, sleep duration), job posture, and transportation tools.^[2,3]^ Spinal disorders, especially CS is a degenerative disorder that become an increasingly important social and medical problem of the modern world, which can significantly decrease the quality of life and affect the normal function of neck and arm.^[2,4]^

### 1.2 Description and function of intervention

Yijinjing is a unique Chinese traditional exercise technique and a synthesizer of traditional Chinese kung Fu. It emphasizes the integrated function of the spine, and pays attention to the coordination of breathing and consciousness during exercise.^[5,6]^ Research found that Yijinjing can make the body temperature appropriately elevate, thus increasing the skeletal muscle stretch and elasticity, and reduce the muscle viscosity, thereby improving the physical characteristics of skeletal muscle.^[7]^ Finally it can improve the strength, flexibility and balance of the muscles and ligaments around the spine, and improve the stability of body, especially the upper and lower limbs and all joints of the spine,^[8,9]^ therefore, it can prevent and cure CS and lumbar disease etc.^[10,11]^ Traditional Chinese Medicine doctors often apply Yijinjing to treat CS, such as Tuotianshi of Nan Shaolin YiJinJing, the 3th posture-weituoxianchu and the 7th posture-Jiuguibamadao.^[12,13]^

### 1.3 Why the review is important

CS increase significantly in terms of hospital incidence rate, and continues to be a serious health problem worldwide. They result in a significant economic burden.^[3]^ The prevalence of CS is high in worldwide,^[2,3]^ furthermore, CS also connected with neck, arm, and forearm pain, which can significantly decrease the quality of life. In Chinese clinical trials, many treatments for people with CS have drawn the method of Yijinjing.^[12–17]^ Owing to nonstandard measurement, nonuniformed outcomes, subjectivity judgment and other factors, the evidence was still limited. Furthermore, no relevant review or protocol has been published. As a consequence, it is necessary to conduct evidence-based review to evaluate the efficacy and safety of Tuina for acute bronchitis in children. It is urgently needed to accomplish this review.

## 2 Methods

This systematic review has been registered in the PROSPERO network (No. CRD 42020164706). All steps of this systematic review will be performed according to the Cochrane Handbook (5.2.0).

### 2.1 Selection criteria

#### 2.1.1 Types of studies

Randomized controlled trial (RCT) and blinded research will be included. Published clinical trials that reported the efficacy and safety on Yijinjing for patients with CS will be included. RCTs that involve at least 1 Yijinjing related treatment to CS, and 1 control treatment (or blank treatment) will be included. As there is a risk of interference with the outcome, nonrandomized controlled trials will be excluded. Studies of animal experiment, review, case report, and meta-analysis will be excluded.

#### 2.1.2 Types of patients

Patients who were diagnosed as CS, aged 18 to 55 years, will be included, without limits on gender, race, nationality and medical units.

#### 2.1.3 Types of interventions and comparisons

Interventions can be any type of Yijinjing. Multiple control interventions will be included: no treatment, placebo and other interventions (e.g., acupuncture, cupping therapy, drugs and physical interventions, moxibustion). If its interventions and comparisons both contain Yijinjing, the study will be excluded. Interventions of Yijinjing combined with other therapies will be included, only if these combinations are compared to the other therapies semplice.

#### 2.1.4 Types of outcomes

Main outcomes will include the neck disability index (NDI), neck pain questionnaire questionnaires. NDI consists of 10 items, including: neck pain and related symptoms (pain intensity, headache, concentration and sleep) and daily living and mobility (personal care, lifting heavy objects, reading, working, driving and playing). Each item was rated from 0 to 5 points. The NDI index was calculated according to the formula, and the range of NDI index was 0 (no disability) to 100% (total disability). The higher the index was, the more serious the dysfunction was. NDI is widely used to assess the functional status and treatment effect of patients with various types of CS, with high reliability and validity. MPQ is used to assess the quality of life of patients with CS. The scale investigated the general conditions of patients and described their own pain. Additional outcomes will include Patient Satisfaction Scale and adverse reactions.

### 2.2 Search methods for identification of studies

#### 2.2.1 Electronic searches

The following electronic databases will be searched from the respective dates of database inception to June 1st, 2020: The Cochrane Library, Web of Science, the World Health Organization International Clinical Trials Registry Platform, Springer, EMBASE, MEDLINE, China National Knowledge Infrastructure, the Chinese Biomedical Literature Database, Wanfang database, the Chinese Scientific Journal Database, and other sources. All published randomized controlled trials (RCTs) about this subject will be included. Exemplary search strategy of MEDLINE is listed in Table 1, terms are conformed to medical subject heading. According to the different retrieval modes, keywords may combine with free words and comprehensive search will be performed.

**Table 1:**
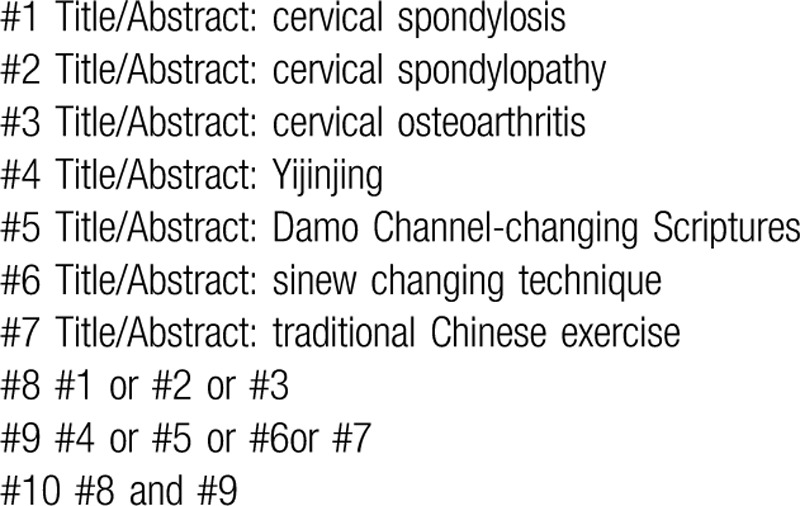
MEDLINE search strategy.

### 2.3 Data collection and analysis

#### 2.3.1 Selection of literature

**Figure 1:**
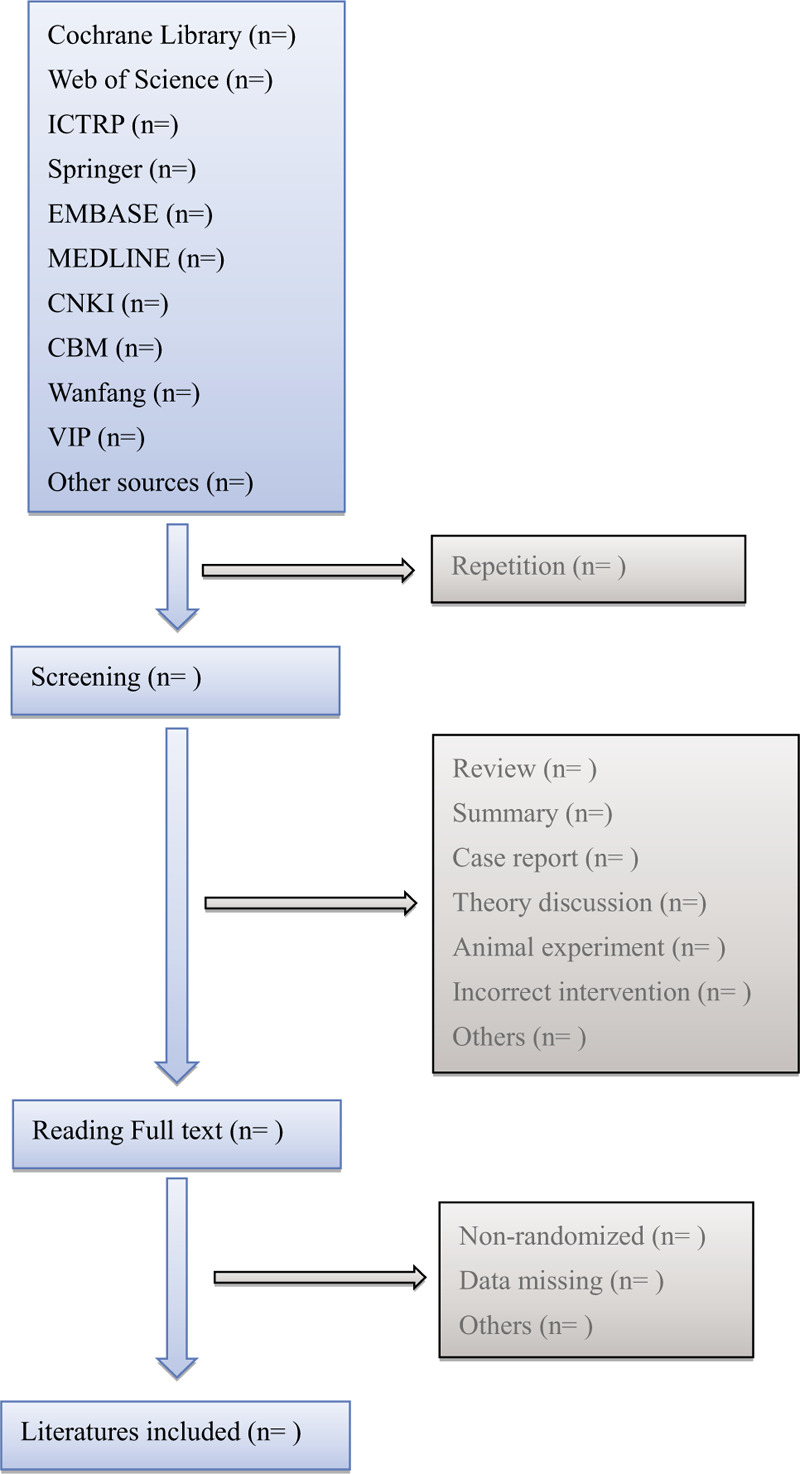
Flow diagram of literature retrieval.

Two authors (QZ and JR) will select clinical trials conforming to inclusion criteria independently. After the articles are screened, disrelated, repetitive, non-standard literatures will be excluded. Screening operation will be rendered in Figure 1. If the full literatures are unable to obtain or related data is incomplete, we will contact the corresponding author. Third-party experts will be consulted to determine the selection divergence.

#### 2.3.2 Assessment and quality of included literature

Two independent authors (FJ and HF) will evaluate quality of included literature and assess the risk of bias by Review Manager (5.3.5) based on Cochrane Handbook (5.2.0). Quality will be assessed from five aspects: randomized method, allocation concealment, blinding methods (participants, personnel, and outcome), completeness of outcome data and selective reporting. Third-party experts will be consulted to determine the selection divergence.

#### 2.3.3 Data extraction

Two independent authors (HF and FJ) will extract the data from the articles selected for inclusion, and resolve differences in opinion through discussion with experts. Data will be recorded onto an electronic form, including categories for basic information about the studies (numbering, the first author's last name and the year the study was published, and the contact information for the corresponding author), the sample sizes and grouping methods used, participant characteristics including age and gender, expressed as mean additions and subtractions above and below standard deviation and the percentages, and details of the intervention methods involved, including treatment time, the selection of posture, treatment efficacy, treatment cycles, side effects, and follow-up.

#### 2.3.4 Measures of treatment effect

Two authors (QZ and YL) will analysis independently and cross-check treatment effect by Review Manager 5.3.5. Risk ratio (RR) with 95% confidence intervals (CIs) will be adopted if dichotomous data exists. Continuous data will be presented by mean difference or standard mean difference with 95% CI. Other binary data will be changed into the RR form for analysis.

#### 2.3.5 Dealing with missing data

As the necessary data in the literature may be lack, we will contact the corresponding authors by email or other contacts. If the missing data is unavailable, we will analysis the existing data and suppose the missing data as random missing.

#### 2.3.6 Assessment of heterogeneity

The heterogeneity of studies will be evaluated by Q-test and I^2^ statistic with RevMan5.3.5. The following criteria will be used: *I*^*2*^ < 50% will be deemed as low heterogeneity; *I*^*2*^ between 50% and 75% will be considered as moderate heterogeneity; *I*^*2*^ > 75% will be considered as high heterogeneity.

#### 2.3.7 Assessment of reporting bias

Funnel plots will be created to assess the reporting bias. Dissymmetry funnel plot indicates high risk of reporting bias, while symmetric funnel plot indicates low risk.

#### 2.3.8 Data synthesis

A meta-analysis or descriptive analysis will be carried out based on measurement methods, intervention methods, heterogeneity levels, etc. If clinical and methodological heterogeneity are low, the fixed-effect model will be applied by merger analysis; the random-effects model will be applied by merger analysis when heterogeneity indicates a moderate level. If a significant level of heterogeneity is found, a descriptive analysis will be performed instead.

#### 2.3.9 Subgroup analysis

Subgroup analysis will be performed based on the findings from the data synthesis. If the heterogeneity is found to have been caused by particular features of the included studies (e.g., the intervention methods [type, time, and cycle] and the measurement methods used in the clinical trials), subgroup analysis will be conducted relevant to these categories.

## 3 Discussion

CS has a high incidence in modern society. Surgical treatment or drug treatment is not necessary in many cases. As a noninvasive external physiotherapy, Yijinjing is widely used for CS in China with the characteristics of simple, convenience, low cost, etc. In recent years, more and more clinical reports on the treatment of CS, but high quality trail is still insufficient. This review will begin when necessary trails are meeting. In order to give compelling evidence and better guide in clinic practice, all actions of this review will be performed according to Cochrane Handbook 5.2.0.

## Author contributions

**Conceptualization:** Qian Zhuang, Fujie Jing, Jing Zhang, Huichao Feng.

**Data curation:** Huichao Feng, Jiao Rong, Fushi Jing.

**Investigation:** Jing Zhang, Yueming Lv.

**Methodology:** Qian Zhuang, Huichao Feng.

**Supervision**: Huichao Feng, Jiao Rong.

**Validation:** Fujie Jing, Jing Zhang.

**Visualization:** Qian Zhuang.

**Writing – original draft:** Qian Zhuang, Huichao Feng, Yueming Lv.

**Writing – review & editing:** Fujie Jing, Jing Zhang.
